# A Geriatric Nurse-led Callback System to Reduce Emergency Department Revisits in Older Adults

**DOI:** 10.5811/westjem.47054

**Published:** 2025-11-18

**Authors:** Jennifer Roh, Luke Walls-Smith, Salman Mushtaq, Luis Gonzalez, Valencia Giles, Lindsey Spiegelman, Soheil Sadaat

**Affiliations:** * Harbor-UCLA Medical Center, Department of Emergency Medicine, West Carson, California; † University of California Irvine Health, Department of Emergency Medicine, Orange, California; ‡ University of California, Irvine Medical Center, Orange, California

## Abstract

**Introduction:**

Emergency departments (ED) present unique challenges for elderly patients who often experience higher revisit rates, increased number of complications, and worse health outcomes. This study examines the impact of implementing a combined automated screening callback and Geriatric Emergency Nurse Initiative Expert (GENIE)-led callback system on reducing ED revisit rates among elderly patients.

**Methods:**

We conducted a retrospective analysis that compared revisit rates before and after the implementation of a GENIE callback system in the ED of a large, Level 1 trauma academic center. The study cohort included 23,664 patients, and the primary outcome was revisits at three, seven, and 30 days post-discharge from the ED. Data were adjusted for the Emergency Severity Index (ESI), age group, and sex. The cost of this initiative came from a three-year grant of $650,000 from the Gary and Mary West Foundation, which included the salary for a GENIE nurse.

**Results:**

Revisit ratios in the pre-intervention period were 4.8%, 8.9%, and 17.2% at three, seven, and 30 days after discharge, respectively. Following implementation of the callback system, those ratios decreased to 3.9%, 7.6%, and 15.2% at the corresponding time points. All reductions were statistically significant (P < .001) and remained significant after adjusting for ESI, age group, and sex.

**Conclusion:**

The GENIE callback system effectively reduced ED revisits among elderly patients, highlighting the importance of structured follow-up communication and care. These findings support the expansion of such programs to improve patient outcomes and reduce healthcare costs.

## INTRODUCTION

Emergency departments (ED) are critical access points for healthcare; however, the environment, system, and overall experience can be particularly challenging for elderly patients. Elderly patients often have complex health needs, leading to longer stays, increased number of complications, and higher revisit rates. These repeat visits not only burden the healthcare system but also indicate potential gaps in patient care and follow-up. In response, a large, academic medical center partnered with a company that created a customized callback system using prerecorded voice actors speaking the patient’s native language, as well as a phone tree including a Geriatric Emergency Nurse Initiative Expert (GENIE) to follow up with all elderly patients post-discharge from the ED. In this study we aimed to evaluate the impact of this system on reducing all-cause ED revisit rates among elderly patients.

Current data indicate that older adults, defined as those ≥ 65 years of age, represent approximately 19.4% of all ED visits in the United States, amounting to approximately 27 million annual visits.^1^ As demographic trends continue to shift toward an aging population, it is projected that by 2060, one in four Americans will be an older adult, with the population of those ≥ 65 years of age expected to double from 2016 figures, and those ≥ 85 projected to triple.^2^

Older adults present unique challenges for EDs, as they tend to use emergency services more frequently than younger demographics, have higher urgency levels during visits, stay longer in the ED, are more likely to be admitted or to revisit the ED, and experience higher rates of adverse health outcomes post-discharge.^3^ A review of patient comments from before and after the COVID-19 pandemic highlighted that negative feedback regarding ED visits primarily centered on communication issues between healthcare practitioners across the care continuum and the professionalism of ED personnel.^4^

To optimize care for older adults in the ED, we implemented a multidisciplinary group of nursing and ancillary staff services following the geriatric emergency department initiative of the American College of Emergency Physicians. A similar program has demonstrated success in increasing the likelihood of older adults being discharged from the ED without increasing the risk of returning to the ED for the same reason, or mortality.^5^ We aimed to further minimize revisits among this demographic with a combination callback program, focusing on personalized post-discharge interventions.

Research conducted by Fruhan et al showed that employing an automated telephone call two days post-discharge significantly decreased revisits within a seven-day period.^6^ Despite the promising results, this study was limited by a relatively small enrollment of 8,110 patients and a focus on a seven-day revisit period. We aimed to build on these findings by investigating the relationship between an automated callback system, enhanced by a dedicated emergency nurse with specialized geriatric training and education, and revisit rates at three, seven, and 30 days post-discharge.

## METHODS

The GENIE callback system was implemented on June 14, 2021. We included patients ≥ 65 years of age who visited the ED between January 2019–June 2021 (pre-intervention) and September 2021–May 2023 (post intervention), covering a period of 30 months prior to and 20 months following the implementation of the GENIE callback system (the intervention). All ED patients ≥ 65 of age who were discharged home from the ED and had not been admitted to the hospital received an automated telephone follow-up call within 24 hours of discharge. The automated system used a standardized structured script to assess the patient’s post-discharge status and identify potential concerns requiring further intervention. Patients received calls in their preferred language (English, Spanish, Vietnamese, Mandarin, or Korean) via prerecorded voice actors reading from a script.

**Population Health Research Capsule**

What do we already know about this issue?

*Older adults experience high emergency department revisit rates due to complex needs; post-discharge follow-up may reduce these visits.*

What was the research question?

*Can a technology-supported, nurse-led callback system reduce ED revisit rates among discharged older adults?*

What was the major finding of the study? *Emergency department revisits dropped at 3, 7, and 30 days (4.8→3.9%, 8.9→7.6%, 17.2→15.2%; all P < .001).*

How does this improve population health?

*Targeted follow-up for older adults reduces the number of preventable ED revisits, easing system strain and improving continuity of care.*

The call included three primary questions regarding symptoms, discharge instructions, and medication access. If patients were satisfied with their care and had no questions, no referral for an additional follow-up call was created. If the patient stated that their symptoms were worse, that response triggered a prompt advising the patient to call 9-1-1 if needed and generated a referral to the GENIE callback queue for a follow-up call if the patient had questions regarding their discharge instructions or had multiple questions. If the patient only had a medication-related concern those calls were routed to an ED pharmacist. However, if the patient responded that they had a medication question and additional questions, their case was also referred to the GENIE callback que for a follow-up call. The GENIE nurse reviewed the callback queue and returned patients’ calls to address their questions. The GENIE callbacks were conducted without a prescriptive script, allowing for personalized follow-up care based on the patient’s reported issues.

The GENIE performed these callbacks during her ED shifts, Monday through Thursday 8 am-5 pm and Friday 8 am-noon. We extracted data from retrospective review of the electronic health records. Revisits were defined as a return visit to the ED within three, seven, and 30 days post-discharge. We excluded from analysis records with missing data, and no imputation was performed. The following elements of optimal chart review as defined by Woster and Bledsoe were followed for this study: case selection criteria; variable definition; medical record identification; sampling method missing data management; and obtaining institutional review board approval.^8^

Patients were stratified into two age groups: 65–74; and ≥ 75 years of age. We compared the proportion of revisits at three, seven, and 30 days post-discharge before and after the intervention using the Pearson chi-square test. We subsequently used a generalized linear model to compare revisit rates pre- and post-intervention, adjusting for age group, sex, and Emergency Severity Index (ESI). Data were analyzed using SPSS Statistics for Windows v29.0.2.0 (IBM Corporation, Armonk, NY), with a type I error level set at 5%.

The callback system was part of a much larger initiative to develop an accredited comprehensive geriatric ED program, which was grant-funded by the West Health Institute (Gary and Mary West Foundation, La Jolla, CA). The total amount of grant funding for this project was $647,167. The GENIE nurse’s salary was covered by the grant for three years (approximately $87,516 per year), and the hospital covered her benefits (total salary was approximately $132,870 per year). The hospital funded subscription to an automated callback system (Cipherhealth, New York, NY). Thus, most grant funding for the callback project went toward the GENIE nurse’s salary. (Of note, the GENIE nurse had multiple other functions in addition to the callback program.) The GENIE role was eventually approved to be a full-time role in the ED, and the ED funded her position in full after the grant ended. During the study period, other experienced nurses would fill the GENIE callback role in the absence of the GENIE nurse.

The GENIE nurse documented a range of follow-up interventions during patient callbacks, which were categorized into five main themes. The most frequent intervention involved general status assessments, including checking on the patient’s condition, providing education or clarification, and assessing pain or other symptoms. A significant portion of follow-up efforts involved assisting patients with follow-up appointments by coordinating care with appropriate clinics or clinicians. A third intervention included clarification of discharge instructions—reviewing written information, lab results, and actions to take if symptoms worsened. The GENIE also addressed issues related to obtaining prescriptions, including contacting pharmacies or helping patients overcome access barriers. Finally, a smaller subset of interactions involved answering medication-related questions, such as how to take medications or understanding potential side effects. These themes highlight the GENIE’s role in bridging care transitions and addressing common gaps in post-discharge understanding and support.

The total number of calls initiated by the automated system was 9,824. Of those calls, 8,842 patients were reached by the automated callback system, and 5,738 (65.0%) responded to at least one callback question. Among these, 1,416 (24.7%) patients identified an issue requiring follow-up. The GENIE nurse followed up on and closed 1,149 of these issues, representing 81.2% of all issues flagged by patients. This accounts for 20.0% of all patients who engaged with the system and 13.0% of all patients reached by the automated calls. Of the 1,149 issues closed by the GENIE, 648 (56.4%) included detailed documentation of the resolution in the medical record.

## RESULTS

The study included a total of 23,664 patients, comprising 12,173 patients in the pre-intervention group and 11,491 patients in the post-intervention group (Table 1). The proportion of revisits observed during the pre-intervention period was 4.8%, 8.9%, and 17.2% at three, seven, and 30 days post-discharge, respectively. In the post-intervention period, these proportions were 3.9%, 7.6%, and 15.2% at the corresponding time points. The differences observed between the pre- and post-intervention periods were statistically significant across all three timeframes (*P* < .001) (Table 2).

**Figure 1:**
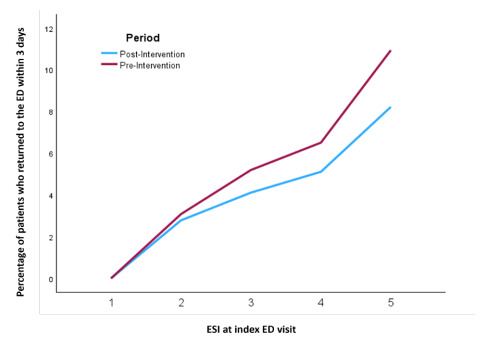
Revisits at 72 hours before and after the geriatric callback intervention, per Emergency Severity Index. *ED*, emergency department; *ESI*, Emergency Severity Index.

Figure 1 illustrates revisits within three days by Emergency Severity Index (ESI) before and after the intervention. The number of revisits increased by higher ESI in both the pre- and post-intervention periods. Furthermore, the disparity in revisits between the pre- and post-intervention periods was more pronounced at higher ESI levels.

In the multivariable analysis, revisits within three days post-discharge (Table 2) were higher during the pre-intervention period (*P* < .001). It was also associated with male sex (*P* < .001), younger age (*P* = .05), and a higher ESI (*P* < .001).

Similarly, revisits rates at seven and 30 days post-discharge were significantly higher during the pre-intervention period (*P* < .001) and were associated with male sex (*P* < .001), younger age (P < .05), and higher ESI scores (*P* < .001) in multivariable analysis.

## DISCUSSION

The findings of this study demonstrate that the GENIE callback system significantly reduced revisit rates among elderly patients at three, seven, and 30 days post-discharge. Importantly, this reduction remained significant even after adjusting for several confounding variables, including ESI, age group, and sex. Elderly patients are disproportionately affected by high revisit rates, a multifactorial issue often driven by chronic comorbidities, polypharmacy, and insufficient social support. The GENIE callback system addresses these challenges by providing structured, personalized, follow-up care post-discharge. This approach ensures timely identification and resolution of potential complications, enhances adherence to discharge instructions and medication regimens, and offers direct access to a dedicated expert for addressing patient concerns.

A key strength of the system lies in its unique design, which designates a specialized geriatric nurse to lead the follow-up process. This proactive and targeted approach likely accounts for the intervention’s success. While specialized ED geriatric nurses have been used in other contexts with positive outcomes,^5^ this study is the first to evaluate their impact specifically on post-discharge revisits in elderly ED patients. The results emphasize the critical role of the GENIE in bridging gaps in care and reducing avoidable ED visits. Our findings also highlight the broader implications of follow-up interventions for healthcare systems. By reducing revisits, the GENIE callback system aids in alleviating the burden on emergency services. This is particularly significant given the increasing demand for emergency care and the associated strain on healthcare resources. Programs like the GENIE callback system could play a pivotal role in addressing these systemic challenges while enhancing the quality of care for an aging population.[Table t3-wjem-26-1738]

## LIMITATIONS

The most significant limitation of this study is its before-and-after design, which meant that we were unable to account for factors such as the COVID-19 pandemic. Additionally, although we adjusted for several confounders, residual confounding variables such as socioeconomic status, caregiver support, non-ED or external healthcare system access, and specific comorbidities may have influenced the observed outcomes. Additionally, this study was conducted in a single academic healthcare setting, potentially limiting the generalizability of the findings to other healthcare systems or community hospitals.

Another possible confounding factor was that our institution established a geriatric ED program in 2019 and achieved Level 1 Geriatric Emergency Department Accreditation (GEDA) on March 7, 2022. Although during that period the GENIE nurse did not formally call back patients in a systematic way, the GENIE nurse worked in the ED and the GEDA program was in effect prior to the implementation of the callback system June 14, 2021. To our knowledge, no significant number of callbacks were made by the GENIE. Additionally, there was no formal system in place to follow up with ED patients beyond the usual clinical care provided by physicians and advanced practice clinicians, which included answering questions if patients initiated calls to the ED after their initial visit. A final study limitation is that our GENIE nurse worked Monday through Thursday 8 am–5 pm and Friday 8 am-noon. Patients who were discharged on Friday and over the weekend were called back on Monday. This gap in callbacks could potentially have led to increased revisits on days when callbacks were not occurring.

Future research should explore the cost-effectiveness of the GENIE callback system, as the financial feasibility of scaling such programs is a critical consideration. In addition, it would be helpful if future studies evaluated the impact on revisits if GENIE coverage was seven days a week instead of five. Furthermore, qualitative studies capturing patient and caregiver experiences with the system could provide nuanced insights into its strengths and areas for improvement. Subgroup analyses examining the system’s impact on patients with specific conditions, such as dementia or heart failure, could also help tailor interventions for maximum effectiveness. Additional studies could also investigate specific impacts on patient health outcomes and patient satisfaction related to curated callbacks post initial ED visits.

## CONCLUSION

The implementation of the Geriatric Emergency Nurse Initiative Expert callback system significantly reduced revisits rates among elderly patients post-ED discharge, highlighting its potential as an effective follow-up intervention. This study provides evidence supporting the broader adoption of specialized, nurse-led and technology-supported programs to enhance care quality, improve patient outcomes, and mitigate pressures on overburdened emergency departments. Future work focusing on long-term benefits, sustainability, and cost-effectiveness will be essential in guiding the integration of such initiatives into comprehensive care strategies for older patients.

## Figures and Tables

**Table 1:** Themes and queries of callback issues addressed by the Geriatric Emergency Nurse Initiative Expert.

Post-discharge issues addressed by the GENIE	Subcategory/description
General status checks	Giving encouragement or general patient check-in
	Assessing pain
	Providing reassurance
Follow-up appointment assistance	Helping schedule follow-up care
	Directing transfer to the correct department or clinic
	Coordinating with primary care physicians
Clarification of discharge instructions	Reviewing written instructions
	Reviewing lab results
	Discussing next steps if symptoms worsened
Obtaining prescriptions	Ensuring prescriptions were filled
	Contacting pharmacies
	Addressing access barriers
Medication-related questions	Explaining how and when to take medications
	Discussing side effects or drug interactions

*GENIE*, Geriatric Emergency Nurse Initiative Expert.

**Table 2:** Comparison of patient population and revisit ratios before and after implementing a geriatric callback program.

		Study period			

		Pre-intervention		Post-intervention	
	
		Count	Column %	Count	Column %
Age group at the index ED visit	> 75	5,759	47.3%	5,544	48.2%
	65–74	6,414	52.7%	5,947	51.8%
Sex	Female	6,537	53.7%	6,148	53.5%
	Male	5,636	46.3%	5,343	46.5%
Patient returned to the ED within 3 days	No	11,583	95.2%	11,047	96.1%
	Yes	590	4.8%	444	3.9%
Patient returned to the ED within 7 days	No	11,093	91.1%	10,617	92.4%
	Yes	1,080	8.9%	874	7.6%
Patient returned to the ED within 30 days	No	10,079	82.8%	9,749	84.8%
	Yes	2,094	17.2%	1,742	15.2%

*ED*, emergency department.

**Table 3 t3-wjem-26-1738:** Generalized linear model analysis of variables associated with 3-, 7-, and 30-day post-discharge revisits.

Post-discharge Interval	Independent variable (predictor)	B	SE	Hypothesis test		

				Wald χ^2^	df	P-value
3 days	Intercept	−4.31	.161	720.60		
	Post- vs pre-intervention period	−.23	.065	12.24	1	< .001
	Age group: (65–74 vs ≥ 75)	.13	.065	4.03	1	.05
	Sex: male vs female	.44	.065	45.41	1	< .001
	ESI	.35	.050	49.49	1	< .001
7 days	Intercept	−3.49	.119	862.25		
	Post- vs pre-intervention period	−.16	.048	11.06	1	< .001
	Age group: (65–74 vs ≥ 75)	.10	.048	862.25	1	< .001
	Sex: male vs. female	.36	.048	58.02	1	< .001
	ESI	.32	.038	71.20	1	< .001
30 days	Intercept	−2.62	.089	870.77		
	Post- vs pre-intervention period	−.14	.038	16.24	1	< .001
	Age group: (6574 vs ≥ 75)	.11	.036	8.95	1	< .001
	Sex: male vs female	.32	.036	79.03	1	< .001
	ESI	.29	.028	102.32	1	< .001

*SE*, standard error; *df*, degrees of freedom; *ESI*, Emergency Severity Index.
